# Online Health Monitoring using Facebook Advertisement Audience Estimates in the United States: Evaluation Study

**DOI:** 10.2196/publichealth.7217

**Published:** 2018-03-28

**Authors:** Yelena Mejova, Ingmar Weber, Luis Fernandez-Luque

**Affiliations:** ^1^ Qatar Computing Research Institute Hamad Bin Khalifa University Doha Qatar

**Keywords:** social media, public health, Internet, infodemiology

## Abstract

**Background:**

Facebook, the most popular social network with over one billion daily users, provides rich opportunities for its use in the health domain. Though much of Facebook’s data are not available to outsiders, the company provides a tool for estimating the audience of Facebook advertisements, which includes aggregated information on the demographics and interests, such as weight loss or dieting, of Facebook users. This paper explores the potential uses of Facebook ad audience estimates for eHealth by studying the following: (1) for what type of health conditions prevalence estimates can be obtained via social media and (2) what type of *marker interests* are useful in obtaining such estimates, which can then be used for recruitment within online health interventions.

**Objective:**

The objective of this study was to understand the limitations and capabilities of using Facebook ad audience estimates for public health monitoring and as a recruitment tool for eHealth interventions.

**Methods:**

We use the Facebook Marketing application programming interface to correlate estimated sizes of audiences having health-related interests with public health data. Using several study cases, we identify both potential benefits and challenges in using this tool.

**Results:**

We find several limitations in using Facebook ad audience estimates, for example, using *placebo* interest estimates to control for background level of user activity on the platform. Some Facebook interests such as *plus-size clothing* show encouraging levels of correlation (*r*=.74) across the 50 US states; however, we also sometimes find substantial correlations with the placebo interests such as *r*=.68 between interest in *Technology* and Obesity prevalence. Furthermore, we find demographic-specific peculiarities in the interests on health-related topics.

**Conclusions:**

Facebook’s advertising platform provides aggregate data for more than 190 million US adults. We show how disease-specific *marker interests* can be used to model prevalence rates in a simple and intuitive manner. However, we also illustrate that building effective marker interests involves some trial-and-error, as many details about Facebook’s *black box* remain opaque.

## Introduction

### Facebook Use in Health Domain

Nearly one third of the world population is using social media and the Internet for entertainment, study, work, and socializing. Currently, Facebook is the most popular social network, with over 1.7 billion monthly active users (as of the end of 2016). Due to this popularity, many health organizations, including hospitals, governments, and patients associations, use Facebook as a channel for health communication [1]. For example, a study by Griffi et al found that over 90% of the US Medicaid/Medicare hospitals had Facebook accounts [2].

Since as early as 2008, there has been interest in the health domain concerning the use of Facebook. For example, at the time Parslow highlighted that among the 60 million users, there were many medical students using the social network as a channel for medical education [3]. On the other hand, Ybarra et al found that teenagers shared unhealthy risk behaviors such as unwanted sexual solicitation on Facebook [4].

Since these early studies, the interest in Facebook within the health domain has continued to grow, not only due to the increase in Facebook’s reach but also due to new features of the platform, which include the development of social games [5,6] and apps [7,8]. Over the last decade, Facebook has been used for medical education [9], patient education [10], peer-to-peer support, organ donation promotion [7], hospital quality estimation [11], and health policy making [12]. Overall, the 2 most popular use cases of Facebook in the health domain, as explained below, are for recruitment and health communication, and public health monitoring. Increasingly, both of these practices rely on the use of Facebook Advertising platform, as we also explain below.

### Facebook for Recruitment and Health Communication

One of the main advantages of Facebook’s popularity is the possibility of using it for the recruitment of people affected by not-so-common conditions such as auditory hallucinations [13]. It can also be used for targeted recruitment of people with particular demographic profiles [14-16] or health behavior (eg, long-term smoking [17]). This can be done by interacting with different Facebook groups [15] or via targeted advertisement. Furthermore, many health care organizations are using Facebook for communication with health consumers. For example, hospitals use Facebook to increase awareness about health-related topics and also to communicate with their patients [2]. Public health administrations also use Facebook to raise awareness about important topics, such as smoking cessation [18], organ donation [7], newborn screening [19], and health education [20]. Furthermore, this communication from public health authorities can be used as mechanisms for health policy making [12] and notifying people at risk of infectious diseases [21].

### Social Media as a Health Tracking Tool

The study of new social media data sources to understand health interests and behaviors is a crucial part of infodemiology [22]. Indeed, social media has been widely used by researchers to study health trends, such as those in health care facility usage [23], abortion information seeking [24], outbreak detection [25], vaccine hesitancy [26], and others. Studies have also found that using social media for seasonal flu tracking outperforms the use of Google search logs for this purpose [27,28] as social media provides more context about why a term is used (or searched for), thus reducing false-positive rates. Moreover, mobile advertisement tools provide fine-grained demographics of mobile app users. One of the most popular is Flurry Analytics, which is owned by Yahoo Inc., which has been used to study the demographics of health apps [29,30]. However, the boundary between mobile analytics and Web analytics is becoming increasingly blurry as the usage of online websites is becoming increasingly mobile and social media companies such as Facebook acquire mobile apps such as Instagram or WhatsApp.

### Facebook Advertisements

As an advertising platform, Facebook allows advertisers to selectively show their ads to Facebook users matching certain criteria, specified by the advertiser. Even before launching—and paying for—the ad, Facebook provides estimates of the expected audience size. As an example, one can ask Facebook for the number of users residing in Alabama who are male, aged 25 to 34 years, and who have shown an interest in *Diabetes mellitus awareness* to receive an estimate of 11,000 users. These tools are available for free in the Facebook Adverts Manager [31]. Facebook documentation explains that the interests are determined from “things people share on their Timelines, apps they use, ads they click, Pages they like and other activities on and off of Facebook and Instagram. Interests may also factor in demographics such as age, gender, and location” [32].

A few recent studies have attempted to link what people like on Facebook to behavioral aspects related to health conditions [33,34]. Gittelman et al converted over 30 Facebook *likes* categories to 9 factors to use in the modeling of mortality [35]. Although they show an improvement in the statistical models, their approach avoided determining relationships between each individual category with the real-world data, limiting the insight into the usefulness of each Facebook interest. On the other hand, Chunara e al explored the relationship between 2 factors, namely, interest in television and outdoor activities, and the obesity rates in metros across the United States and neighborhoods within New York City [36]. Although showing promising correlations, the latter study failed to account for baseline user activity, potentially reporting relationships indistinguishable from general Facebook. In this paper, we address the shortcomings of both these studies.

### Study Goals

Previous studies have attempted to demonstrate the value of using Facebook ad audience estimates for modeling regional variations of the prevalence of certain health conditions [35,36]. However, these studies fail to compare the strength of the relationships between Facebook interests and real-world health statistics to *baseline* relationships, potentially reporting spurious results due to the *black box* nature of the tool. In this study, we propose 2 methods for gauging the strength of such relationships: first by introducing *placebo* interests which to a varying extent represent baseline Facebook user behavior, and second by examining alternative normalization populations. Thus, we contribute to the methodological literature addressing the different variables that can affect the use of Facebook interest data for public health monitoring, in an attempt to lessen the barriers for comparison and reproducibility of studies employing such data.

## Methods

### Facebook Advertisement Audience Data Collection

**Figure 1:**
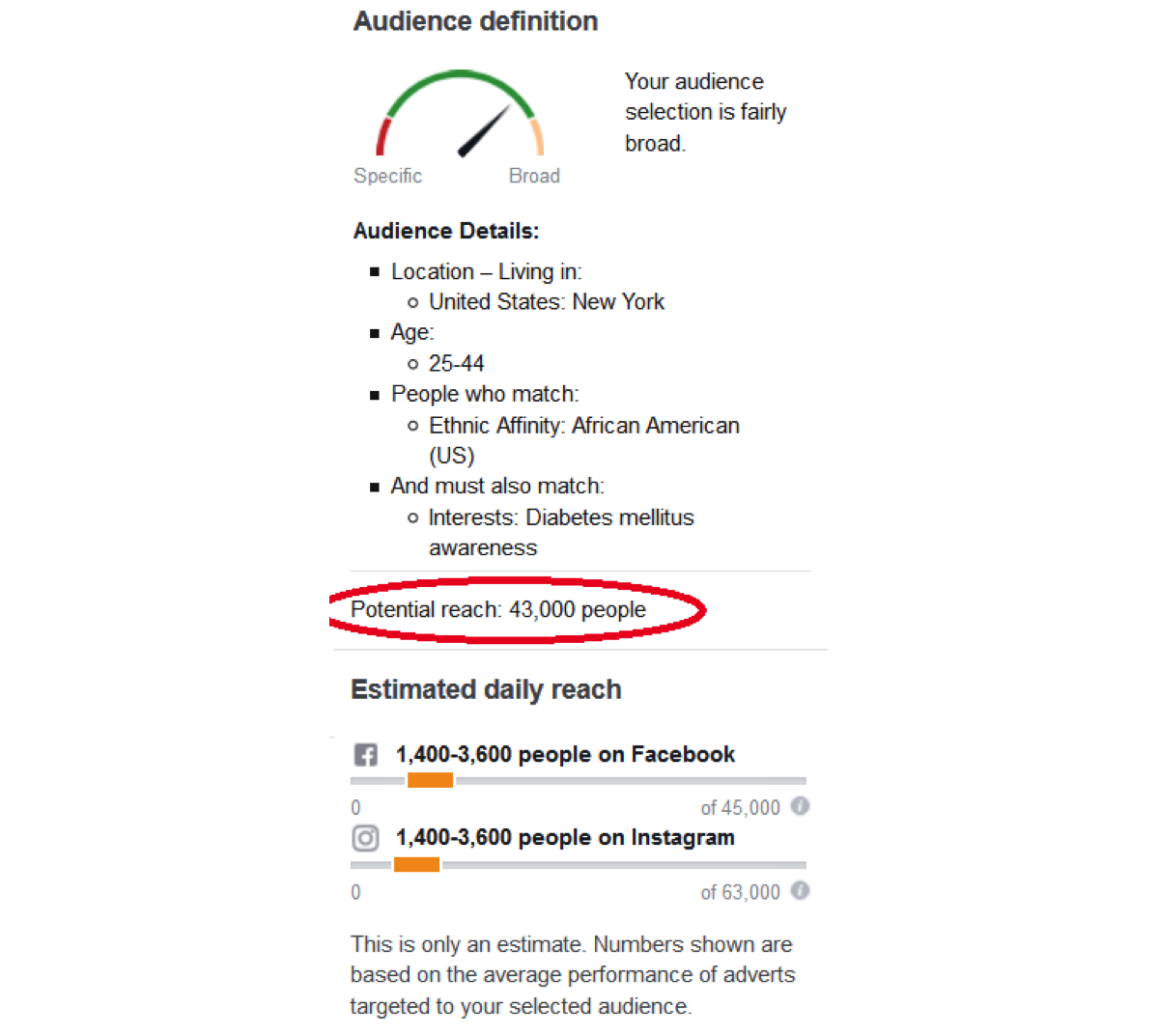
Part of a screenshot of Facebook's Adverts Manager, illustrating some of the targeting capabilities (under "Audience Details") as well as the reach estimate.

All data used for the following analysis are provided by the Facebook’s Marketing application programming interface (API) [37]. Equivalent data could have been obtained through the Web interface of the Adverts Manager, but using the API makes programmatic access easier and gives more precise audience estimates, down to +/-20 users as opposed to +/-1000 users. The numbers we used are the so-called *Reach Estimates*: “Potential reach is the number of monthly active people on Facebook that match the audience you defined through your audience targeting selections” [38]. Figure 1 shows a screenshot of Facebook’s Adverts Manager [31], illustrating the capabilities. As previously defined, Facebook provides an aggregate mapping between users and interests, hiding the source of the data (whether it comes from likes, posts, or other Facebook properties which include Instagram), providing a simplified interface, while also hiding potentially useful information.

For our study, we obtained Facebook data that are potentially related to the prevalence of 4 diverse health conditions: (1) diabetes (type II), (2) obesity, (3) food sensitivities, and (4) alcoholism. As largely behavior-related conditions, these are prominent causes of serious illness and death across the United States. Moreover, they range in the extent of potential social stigma, and their impact on the personal and social life of an individual.

For each of these 4 conditions, we defined a number of *marker interests*. A *marker interest* is an interest of a Facebook user that could plausibly be used to measure the prevalence of a certain condition due to a potential causal link between the condition and the interest.

We used an iterative process to obtain these marker interests—employing domain knowledge, we used the Facebook Adverts Manager interface to exhaustively enumerate interests related to the selected illnesses, selecting all those passing the threshold of US-wide audience in hundreds of thousands. For example, both of the interests *Alcohol* and *Alcoholics Anonymous* are marker interests for alcoholism.

Similarly, we defined a set of *placebo interests*. A *placebo interest* is an interest of a Facebook user that should not have an obvious causal link with a given condition, but that might still turn out to be correlated due to latent factors such as common user demographics.

Placebo interests are helpful to understand how much of any predictive power of marker interests is due to spurious correlations or due to unknown latent factors. Intuitively, these interests are meant as a *placebo* wherein no topic-specific *treatment* is performed, and any effect observed is due to the random or causal factors outside the topic. For this, we used the popular generic interests (ie, *Facebook*, *Reading*, *Entertainment*, *Music*, and *Technology)* that, a priori, should not have any strong link to the 4 conditions studied. Each of these interests is shared by hundreds of millions of Facebook users worldwide, and serves as approximations of the level of involvement of users with the platform in general.

Finally, we also defined a health-related *baseline interest*. A *baseline interest* is a broad health-related interest on Facebook that could plausibly be used to measure general health awareness.

In this study, we used the interest *Fitness and wellness* as a baseline interest. This baseline interest helps to clarify if any predictive power of a marker interest is really due to a condition-specific link to the interest, or if we are only picking up the general health awareness level.

Using these interests, we then queried the Facebook Graph API [39] for the estimation of audience size for each combination of interest and US state, as well as gender (including *both*), age group (18-24, 25-44, 45-64, 65+ years, and all combined), and ethnic affinity (African American, Asian American, Hispanic, none of the above, and all combined). This allows us to look at both correlations across the 50 US states, as well as at correlations across different demographic groups.

On its own, a single audience estimate is of little value. It is only when seen in context that one can judge if a number is high or low. Thus, to normalize the raw audience estimate counts, we defined 3 *reference populations*: (1) number of Facebook users (widest selection), (2) number of users interested in *Facebook* (thus who are more likely to be active on the site), and (3) number of users interested in *Fitness and Wellness* (thus who are more likely to be interested in health-related topics). We then divided the marker and placebo interests by the reference populations, producing 3 variants of proportionate interest measurement. Finally, the Facebook API was queried for the audience estimates in September 2016.

### Public Health Data Collection

The US state-level public health data were obtained via the America’s Health Rankings Annual Report [40], which combines data from well-recognized sources including Centers for Disease Control and Prevention, American Medical Association, Federal Bureau of Investigation, Dartmouth Atlas Project, US Department of Education, and Census Bureau. For our study, we used the most recent available data for 2015 [41]. Data for the District of Columbia were not used, as they had several missing values.

### Comparing Public Health Data and Facebook Advertisements Data

As described above, for each of the 50 states, we have (1) a set of indices derived from Facebook’s ads audience estimates, for example, the fraction of monthly active Facebook users with an interest in the topic *Diabetic Diet*, and (2) a set of public health indices, such as the fraction of the adult population that has diabetes. Each Facebook index *f* consists of a marker, placebo or baseline interest (see definitions above), and a choice of reference population (the set of all Facebook users by default). To see if an index *f* could be used to approximate a particular public health index *h*, we computed the Pearson correlation coefficient *r*_fh_ across the 50 states. Thus, we hypothesized that Facebook indices (independent variables) are related to public health indices (dependent variable). We deliberately chose Pearson *r* for its simplicity and did not experiment with any model fitting, such as multi-variate linear regression, or with non-linear measures of correlation, such as Spearman rank correlation coefficient to clearly show the relationship of each interest, as well as to compare marker interests to the placebos and baselines.

To avoid reporting spurious correlations, we applied a significance threshold of *P*=.05/*k*. Here *k*, the number of experiments performed, is a Bonferroni correction factor to avoid false positives when testing multiple hypotheses. In our setting, each pair of indices *f* and *h* constitutes one hypothesis that is being tested.

### Analyzing Potential Comorbidity

To explore the feasibility of using Facebook data to discover comorbidity, where suffering from one condition increases the probability of suffering from another, we choose *Fatigue* as a target condition. Concretely, we explored these relationships by computing the *lift* statistic between fatigue-related marker interests and others which may be associated with them. Lift is often used in association rule mining as a measure for strength of the association between 2 occurrences, normalized by the likelihood of them occurring by random chance, and has the following formula:

lift(A,B)=P(A∩B)/{P(A)×P(B)}

It can intuitively be understood as *P(A|B)/P(A)=P(B|A)/P(B)*, that is, the *lift* in probability of event A (or B) occurring over its baseline probability, given that event B (or A) has occurred. A value greater than 1.0 indicates an increase in conditional probability, whereas a value smaller than 1.0 indicates a decrease.

## Results

### Interest Selection

Table 1 shows the US-wide audience estimates for the selected marker, placebo, and baseline interests. At the bottom, we also show the Facebook audience of US residents who are aged 18 years or older. Recall that to constrain the number of considered interests, we selected only those having at least hundreds of thousands US-wide audience. Indeed, some interests, such as *Alcoholic beverages* (at 74 million), span a great deal of US Facebook users (totaling at 194 million users, as listed at the bottom of the table). A bootstrapping approach was taken to these, whereby we began with a keyword relevant to the topic (such as *alcohol* for *alcoholism*), and added other related interests, which the Facebook Advertisement Marketing interface provides. Thus, the selection of the interests was *seeded* by domain expertise, and expanded via internal Facebook usage statistics.

### Relation to Public Health Data

**Figure 2:**
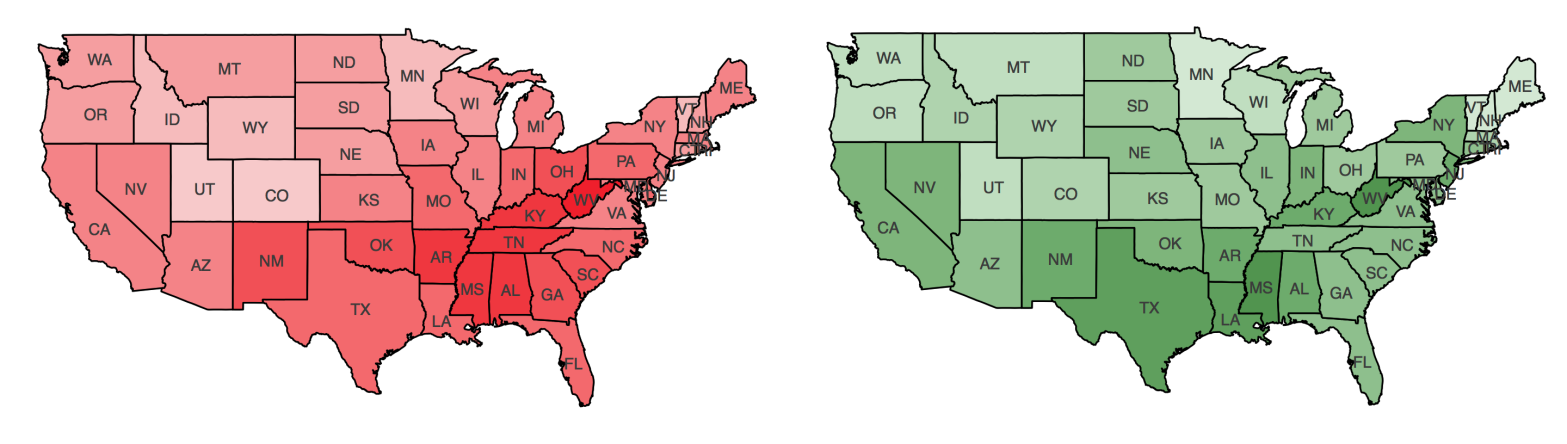
Geographic distribution of (left) diabetes prevalence and (right) percentages for the marker interest "Diabetes mellitus awareness" normalized by FB_pop_, where color saturation represents strength of the variable.

We began with a question—how much do the populations having particular *interests* in health-related topics, as determined by Facebook, correlate with ground truth statistics gathered by Centers for Disease Control and Prevention (CDC)? For visual examination, we plotted the intensities of diabetes prevalence and the percent of interest *Diabetes mellitus awareness* (normalized by the number of Facebook users, *FB*_pop_) in Figure 2. The intense colors in both plots are concentrated in the south, as well as West Virginia, and less so in mountain states as well as Vermont and New Hampshire.

Next, we quantified the relationship between Facebook advertisement audience figures and the ground truth statistics. First, we examined the placebo interests (normalized by *FB*_pop_), as shown in Table 2, along with the accompanying 2-tail significance levels. The health statistics are proportions of the population, including engaging in excessive drinking (results for binge and chronic drinking were similar; hence, we omitted them here), as well as obesity and diabetes rates. Note the strength of the association between some variables, especially obesity and diabetes, with Pearson correlation *r*=.68 between the placebo interest *Technology* and both diabetes and obesity prevalence. Regardless of the forces at play, these figures caution us against considering high *r* values as indicative of causal relationships in the following experiments.

Table 3 shows the correlations of each health-related interest with the appropriate health statistic (eg, between Alcoholism interests and statistics on excessive drinking). The 2-tailed significance tests for these correlations have been adjusted using Bonferroni correction to address the problem of multiple comparisons and guard against false positives. We observe a complex relationship between alcohol-related variables. Although *Alcohol* and *Bars* have little correlation with excessive drinking, *Alcohol abuse* and *Alcoholism awareness* are positively related to it. Interventions, on the other hand, including *Alcoholics Anonymous* and 12 *-step program*, are *negatively* associated with drinking. Note, however, that most values of *r* achieved for Alcoholism are barely larger than the values for the placebo interests *Reading* and *Technology* of around *r*=−.35.

Considering obesity and diabetes, most marker interests are positively correlated with their real-world corresponding statistics, although some correlations vary drastically with the choice of reference population. The strongest and most consistent correlations are between *Plus-size clothing* (*r*=.74) and obesity, as well as *Diabetes mellitus awareness* (*r=*.78) and *Diabetic diet* (*r=*.75) and diabetes.

The variation between correlations across the 3 different reference populations shows that the reference point used for the raw audience counts has strong effects on the results. *Facebook* interest (*FB*_int_) normalization, for instance, removes the effect of users who are in general likely to be active and have interests, some of which by chance may include health-related topics. Similarly, the *Fitness and Wellness* interest (*FW*_int_) removes the effect of general interest in health. As we can see in Table 3, these normalizations affect each interest in a different manner.

Furthermore, we assessed the combined power of these interests in modeling the real-life phenomena by building linear regression models to predict the real-world statistics. As there were only 50 data points in the dataset, we used feature selection using backward feature elimination optimizing Akaike Information Criterion scores, in which least-contributing features were removed until an optimal performance was achieved. The resulting linear models achieve the adjusted *R*^2^ of .533 for modeling Alcoholism, .712 for Obesity, and .790 for Diabetes. Next, we included the following additional control variables: (1) demographics, including age, gender, and race distributions; (2) financial statistics, including median annual household income and unemployment rate; (3) health care-related statistics, including health spending per capita and rate of uninsured persons; (4) internet access rate; and (5) health-related variables, including life expectancy and poor mental health days reported. When applied to this much larger set of variables, the Facebook marker variables were still selected, and the resulting models had an improved performance of .698 (Alcoholism), .827 (Obesity), and .894 (Diabetes). Interestingly, only in the case of Obesity were placebo interests selected for the final models, which were Entertainment and Technology. We discuss the interpretation of these further in the Discussion section.

**Table 1:** List of marker interests: Facebook marker interests for tracking diabetes, obesity, food sensitivities, and alcoholism, along with placebo interests, and a generic health baseline. This table also shows the estimated Facebook audience for US residents aged 18+ years.

Health condition interest		Estimated Facebook audience
**Alcoholism**		
	Alcohol	13,000,000
	Alcohol abuse	610,000
	Alcoholic beverages	74,000,000
	Alcoholics anonymous	670,000
	Alcoholism awareness	4,600,000
	Bars	33,000,000
	Sobriety	3,100,000
	Twelve-step program	640,000
**Obesity**		
	Bariatrics	710,000
	Obesity awareness	7,400,000
	Plus size	430,000
	Plus-size clothing	9,100,000
	Weight loss (fitness and wellness)	15,000,000
	Dieting	27,000,000
**Diabetes**		
	Gestational diabetes	650,000
	Insulin index	250,000
	Insulin resistance awareness	500,000
	Diabetes mellitus awareness	12,000,000
	Diabetes mellitus type 1 awareness	1,200,000
	Diabetes mellitus type 2 awareness	2,100,000
	Diabetic diet	2,100,000
	Diabetic hypoglycemia	230,000
**Food sensitivities**		
	Gluten sensitivity awareness	250,000
	Gluten-free diet	10,000,000
	Lactose intolerance	240,000
	Food allergy	690,000
	Food intolerance	200,000
	Peanut allergy	140,000
**Placebos and baselines**		
	Facebook	83,000,000
	Reading	141,000,000
	Entertainment	171,000,000
	Music	152,000,000
	Technology	157,000,000
	Fitness and wellness	110,000,000
	No interest constraint	194,000,000

**Table 2:** Pearson correlation coefficient *r* between placebo Facebook interest estimates and the US health statistics (normalized by *FB*_pop_ and state population, respectively).

Facebook interest	Health condition					
	Alcoholism		Obesity		Diabetes	
	*r*	*P* value	*r*	*P* value	*r*	*P* value
Reading	−.34^c^	.01	.67^a^	<.001	.58^a^	<.001
Music	−.23	.19	.54^a^	<.001	.59^a^	<.001
Entertainment	−.06	.67	.47^a^	<.001	.24^b^	.09
Technology	−.39^b^	.005	.68^a^	<.001	.68^a^	<.001

^a^*P*<.001.

^b^*P*<.01.

^c^*P*<.05.

### Comorbidities and Related Behaviors

In the previous analysis, we have only considered audience estimates for 1 Facebook interest at a time. However, Facebook’s advertising platform supports the definition of more complex target groups, which express not only those interests that are directly related to the illnesses but also those that indicate behaviors or conditions which may be linked to it. Alcoholism, for example, is associated with depression and anxiety [41], whereas obesity has been linked to poor dietary choices and sedentary lifestyle. As described in the Methods section, we use the notion of *lift* to measure the relationship between 2 interests. It can intuitively be understood as the *lift* in probability of event A (or B) occurring over its baseline probability, given that event B (or A) has occurred. A value greater than 1.0 indicates an increase in conditional probability, whereas a value smaller than 1.0 indicates a decrease.

We selected a variety of interests that may be related to obesity, diabetes, alcoholism, and food sensitivities. Specifically, for the first 2, the interests include physical activities (like *hiking* and *yoga*), nutrition interests (*healthy diet*, *desserts*), specific restaurants (*McDonald’s*, *Subway*), and spectator sports (*NASCAR*). For alcoholism, we included places associated with drinking (*nightclubs*), as well as mental health interests (*mental health*). As the task is exploratory, we did not include all possible related interests, but instead used a selection of 45 having the best Facebook ads audience coverage.

Table 4 shows the 20 marker interests and related interests with greatest lift (that is, which appear more often together than would be predicted by chance), and with smallest lift (which appear less often together than one would observe by chance). Some relationships make sense, such as that between *Alcoholics Anonymous* and *Anxiety Awareness*, as alcoholism is associated with mental health issues. Another example may be *Bariatrics* and *Panera Bread* (a restaurant chain promoted as healthy). However, we caution the reader to impose meaning on these relationships, as these may be caused by other means. For example, the interest *Nightlife* may be highly expressed in urbanized states. Thus, a positive lift might be due to a latent factor, such as urbanization, giving rise to both interests. In future studies it might be worth exploring such alternative explanations by limiting the analysis to urban centers.

### Demographic Exploration

Another potentially powerful feature of Facebook Advertising Manager is the demographic information of its users, including age, gender, and ethnic affinity [42]. We related these to the illness interests in Table 5, similarly listing relationships that are more likely (above) and less likely (below) than one would expect by chance.

**Table 3:** Pearson correlation coefficient *r* between Facebook marker interests and US health statistics, normalized by Facebook users (*FB*_pop_), *Facebook* interest (*FB*_int_), and *Fitness and Wellness* interest (*FW*_int_).

Health condition and corresponding interest		FB_pop_, *r*	FB_int_, *r*	FW_int_, *r*
**Alcoholism**				
	Alcohol	−.17	.08	−.07
	Alcohol abuse	.38	.49^a^	.44
	Alcoholic beverages	−.06	.50^a^	.32
	Alcoholics anonymous	−.36	−.30	−.33
	Alcoholism awareness	.30	.42	.36
	Bars	−.08	.09	.00
	Sobriety	−38	−.26	−.35
	Twelve-step program	−.25	−.07	−.15
**Obesity**				
	Bariatrics	.49^a^	.37	.45^a^
	Obesity awareness	.60^b^	.37	.55^b^
	Plus size	.70^b^	.65^b^	.70^b^
	Plus-size clothing	.74^b^	.72^b^	.75^b^
	Weight loss (fitness and wellness)	.76^b^	−.09	.33
	Dieting	.08	−.34	−.12
**Diabetes**				
	Gestational diabetes	.32	.25	.28
	Insulin index	.58^b^	.43	.53^b^
	Insulin resistance awareness	.52^b^	.39	.46^a^
	Diabetes mellitus awareness	.78^b^	.72^b^	.79^b^
	Diabetes mellitus type 1 awareness	.29	.02	.14
	Diabetes mellitus type 2 awareness	.43	.33	.39
	Diabetic diet	.75^b^	.62^b^	.68^b^
	Diabetic hypoglycemia	.62^b^	.49^a^	.57^b^

^a^*P*<.01.

^b^*P*<.001.

The most powerful relationship is between *Plus size* and *African American* demographic. This relationship is corroborated by the literature on obesity. For instance, according to the US Department of Health and Human Services, “In 2014, African Americans were 1.5 times as likely to be obese as Non-Hispanic Whites” [43]. The association between diabetes and elderly is also supported by CDC, with an estimated 25.9% of the US population aged ≥65 years having diabetes in 2012 [44]. Similarly, the association between diabetes and *Hispanic* demographic is justified by research, with Hispanic adults being 1.7 times more likely than non-Hispanic white adults to have been diagnosed with diabetes by a physician [45].

Some inverse relationships in the right-hand side columns of Table 5 can also be justified by prior literature. Food sensitivities (such as *Lactose intolerance*) are less likely in adult men than women [46]. Similarly, we find a lift of 1.61 between women and *Gluten-free diet*, and women are diagnosed with Celiac disease (hypersensitivity to gluten) 2 to 3 times more often than men [47]. However, these numbers may also show the interests of certain demographics. For instance, it may be that Facebook users over 65 years of age are not interested in *Obesity awareness* or *Diabetes mellitus type 1 awareness* (as the latter is often discovered in children), each having lifts of 0.02. However, not all interpretations are straightforward. Although men are more likely to have diabetes (13.6% males vs 11.2% females have diabetes), they are very unlikely to have an interest in *Insulin index*.

**Table 4:** Most directly related and inversely related illness interests and related interests, as measured using lift.

Illness interest		Related interest	Lift
**Directly related illness interests**			
	Insulin resistance awareness	Nightlife	25.32
	Insulin index	Nightlife	23.12
	Insulin index	Panera Bread	22.40
	Insulin resistance awareness	Panera Bread	22.34
	Bariatrics	Nightlife	21.54
	Bariatrics	Panera Bread	19.93
	Gestational diabetes	Healthy diet	18.90
	Alcoholics anonymous	Anxiety awareness	17.67
	Food intolerance	Healthy diet	17.23
	Insulin index	Mental health	17.19
	Diabetic hypoglycemia	Healthy diet	17.12
	Alcoholism awareness	Anxiety awareness	16.99
	Insulin resistance awareness	Mental health	16.94
	Diabetes mellitus type 2 awareness	Panera Bread	16.22
	Twelve-step program	Anxiety Awareness	16.21
	Major depressive disorder awareness	Nightlife	16.19
	Sobriety	Mental health	16.15
	Food allergy	Anxiety awareness	15.81
	Diabetes mellitus type 1 awareness	Mental health	15.81
	Gluten sensitivity awareness	Mental health	15.81
**Inversely related illness interests**			
	Hepatitis awareness	Nightlife	0.31
	Lactose intolerance	National Football League	0.32
	Hypertension awareness	Fast food restaurants	0.38
	Lactose intolerance	Nightlife	0.42
	Gestational diabetes	Muscle and fitness	0.47
	Food allergy	Fast food restaurants	0.48
	Hepatitis awareness	Dunkin' Donuts	0.49
	Lactose intolerance	Muscle and Fitness	0.51
	Alcoholism awareness	Fast food restaurants	0.54
	Diabetic hypoglycemia	National Football League	0.57
	Diabetic hypoglycemia	Muscle and Fitness	0.57
	Lactose intolerance	Dunkin' Donuts	0.57
	Gestational diabetes	National Football League	0.58
	Alcoholics anonymous	CrossFit	0.58
	Lactose intolerance	Nightclubs	0.60
	Lactose intolerance	Basketball	0.61
	Hypertension awareness	National Football League	0.62
	Diabetic hypoglycemia	Nightlife	0.63
	Lactose intolerance	Fast food restaurants	0.64
	Lactose intolerance	McDonald’s	0.69

**Table 5:** Most directly related and inversely related illness interests and demographics, as measured using lift.

Marker interest		Demographic	Lift
**Directly related illness interests**			
	Plus size	African American	5.38
	Diabetic hypoglycemia	65+ years	3.93
	Diabetic diet	65+ years	3.59
	Insulin resistance awareness	Hispanic	3.48
	Diabetes mellitus awareness	Hispanic	3.01
	Dieting	Hispanic	2.89
	Diabetic hypoglycemia	Asian American	2.86
	Bariatrics	Hispanic	2.71
	Plus-size clothing	African American	2.67
	Diabetes mellitus type 2 awareness	Hispanic	2.60
	Insulin index	Hispanic	2.57
	Obesity awareness	Hispanic	2.57
	Alcohol	Hispanic	2.55
	Diabetic hypoglycemia	Hispanic	2.22
	Bars	Hispanic	2.17
	Lactose intolerance	45-64 years	2.14
	Lactose intolerance	65+ years	2.12
	Alcoholics anonymous	65+ years	2.10
	Insulin index	25-44 years	2.10
	Insulin index	African American	2.06
**Inversely related illness interests**			
	Diabetic hypoglycemia	18-24 years	0.01
	Insulin index	Male	0.01
	Diabetes mellitus type 1 awareness	65+ years	0.02
	Obesity awareness	65+ years	0.02
	Plus size	Male	0.06
	Food allergy	Male	0.07
	Diabetic diet	18-24 years	0.07
	Bariatrics	65+ years	0.08
	Lactose intolerance	Male	0.09
	Alcoholics anonymous	Asian American	0.09
	Gestational diabetes	Male	0.10
	Diabetes mellitus type 1 awareness	45-64 years	0.10
	Alcohol	65+ years	0.11
	Plus size	65+ years	0.11
	Plus-size clothing	65+ years	0.11
	Plus-size clothing	Male	0.11
	Food allergy	18-24 years	0.11
	Gluten sensitivity awareness	65+ years	0.12
	Diabetic hypoglycemia	25-44 years	0.13
	Sobriety	65+ years	0.13

## Discussion

### Methodological Contributions to Using Facebook Advertisement Audience Estimates

To use Facebook advertisement audience estimates for public health is not trivial, as there are many aspects that can affect the interpretability of the data from Facebook. At first, our results seem to confirm previous findings that variations in interests on Facebook across different geographic locations can be used for modeling lifestyle disease prevalence. We were able to find clear correlations of Facebook advertisement audience estimates with available public health data. This is consistent with some of the previous studies published in the literature [14,15,17,35,36]. However, unlike Gittelman et al [35], we examined the contribution of each *marker* interest, and consequently found a variety of behaviors. For example, the performance for *Weight loss* (*r*=.76 for *FB*_pop_) and *Dieting* (*r*=.08 for *FB*_pop_) for modeling obesity rates were vastly different. This means that, as of now, there is a certain amount of trial-and-error involved in finding marker interests that are informative.

Crucially, this work introduces the use of the *placebo interests*, which provides a baseline performance estimate with which the above *marker* interests may be compared. In this study, we show that common topics such as *Reading* and *Technology* can display a nontrivial correlation with ground-truth statistics, making them an important step in verifying the significance of health-specific results. The fact that the interests we have not expected to have a strong relationship with the illnesses have shown substantial correlation may be due to the following: (1) Facebook usage, which may predispose users to certain conditions, (2) a direct relationship between the variables (interest in reading may be associated with a sedentary lifestyle, which is in turn related to diabetes [48]), or (3) some causal relationship via latent factors influencing both variables. Regardless, the strength of the correlations found with these *placebos* stands as a cautionary observation for future social media researchers that *marker* variables need to be interpreted in the light of possible confounding factors.

As a causal explanation may still be at play in an indirect way, the choice of interests that have no relationship with health-related statistics becomes an interesting challenge, as any behavior may have a tangential connection with the lifestyles involved. For instance, during the feature selection process we find Technology and Entertainment being selected to model Obesity (although not Alcoholism or Diabetes). However, if such interests which have no theoretical grounding to be correlated with the disease are found, the extent of their observed relationship with it—as discovered in the data—may provide a glimpse into a *placebo effect* inherent in the data. It is precisely this effect that should determine whether *marker* correlations are strong enough to be considered interesting.

Similar conclusions can be drawn about the exploration of normalization factors. The employment of generic population estimates of Facebook users (*FB*_pop_), compared with general interest in *Facebook* (*FB*_int_), or domain-specific interest in *Fitness and Wellness* (*FW*_int_) all provide a different interpretation of the raw audience estimates, and must be selected according to the aims of the study. Even interests which we found to have lift of 1 could be used as topic-specific placebos. In future work, we plan to design and validate methods to normalize interests across different cohorts. For example, if we know the most common interest among teenagers, we will have a better baseline for gauging interest among teenager for certain interests, such as ultra-caffeinated drinks.

### Challenges of Using Facebook Advertisements as a Black Box

Going forward, the biggest question that one has to address when using Facebook’s advertisement audience estimates for certain interests is the following: what does it *mean* when a certain user has a certain interest as detected by Facebook?

Finding the answer involves understanding 2 different aspects: (1) Facebook's algorithmic black box and (2) Facebook users. On the algorithmic side, Facebook employs a number of classifiers to detect if, for instance, a given user is interested in the topic *Obesity awareness*. The features that go into this classification are largely derived from Facebook pages the user has liked, but also from general Web browsing history (through tracking cookies on pages with Facebook like or share buttons), as well as other information [49]. Understanding this can have important implications for the applicability of this data source for observing stigmatized health conditions where it is less likely that a Facebook user publicly *likes* a page on, for example, genital herpes. However, apart from understanding the importance of various features, there is also the issue of understanding the class labels. What exactly does *Obesity awareness* refer to? And what is the difference between an interest in *Dieting* versus *Weight loss*? Unfortunately, Facebook does not provide the option to show pages labeled with a given interest, or any other way to obtain a better understanding.

But even if one was to perfectly understand the inner workings of Facebook’s classification setup, there still is the fundamental challenge of understanding the user’s inner workings. What does it mean if a user *likes* a page about lung cancer? Has the user been diagnosed with lung cancer? Or someone in their family? Are they just generally concerned about the topic? Having a better understanding of the user’s motivations can lead to a better selection of marker interests. As an example, we observe that an interest in *plus-size clothing* has good predictive power in modeling regional variation in obesity rates. Arguably, this is because having and expressing this interest is closely related to being overweight. However, the same cannot be said for an interest in *Alcohol* and its use to model prevalence of alcoholism. A potential solution to these questions would be to employ the advertisement platform to recruit participants for a survey designed to assess the above questions, and thus evaluate the efficacy of Facebook’s interest inference algorithm. Although research on even smaller regions such as ZIP codes have been performed [50,51], Facebook Advertisement Manager allows for queries focused on even smaller geographical regions—the interface allows for areas as small as 2 km across.

Finally, interests in Facebook can vary longitudinally, both as Facebook’s user base expands and contracts, and as yearly seasonal variations occur. The first change in estimates would explain the general upward trend in the figures reported by this study, as compared with the previous ones [35,36]. The second will require longitudinal tracking and normalization if Facebook advertisement audience estimates are used for monitoring interests over long periods of time. Similarly, such dated information can then be synched with ground truth such as CDC reports for a more precise overlap of the time frame.

### Consequences for Public Health

As explained above, there are limitations in the use of Facebook advertising for public health. We also need to be aware of potential negative consequences of using it. The focus on online sources can exacerbate health disparities due to the heterogeneous levels of digital health literacy [52,53]. If public health stakeholders are relying exclusively on social media data, they might unintentionally leave behind large segments of the population. For example, people with visual impairment might less frequently use social media due to accessibility problems [54].

Furthermore, advanced advertising allows tailoring by interests that are not necessarily health related and can be controversial. For example, it is possible to target people with interest in both cars and alcohol for a campaign to reduce driving under the influence of alcohol. This can be seen by many as a potential violation of privacy. Although users have agreed to the terms of use of social media and mobile apps, often they are not aware of the privacy implications [55]. We strongly advise the development of ethical guidelines and training for conducting health-related studies and interventions in social media. Some of those guidelines already exist, but they require continued updates as the technology evolves [56,57].

This paper, as any health-social media paper, can be also used intentionally as a source of information to do harm [58]. We need to be aware that our research can be used by communities that engage in Facebook to do harm (intentionally or unintentionally), such as promoting anorexia as a lifestyle [59], hampering vaccination efforts [60], or even promoting smoking [61]. This potential challenge should not pose a barrier for research in this area; on the contrary, more research can help identify ways to tackle the misleading use of social media in the health domain.

### Privacy

As this research did not involve human subjects, it did not require approval by an institutional review board. All the information we used was collected via an open API provided by Facebook in the public domain. The data provided are always aggregated and cannot be linked to the individual. The provider of data (Facebook) is not a collaborator in the study here described. Furthermore, the Terms of Use of the platform allows the collection of data so that Facebook can provide services to third party organizations with those data, given that it is deidentified. Finally, as discussed in the Methods section, Facebook API rounds up the aggregate numbers to nearest 20, thus allowing for k-anonymity [62] for individuals within an audience for a query of any specificity. We note here that, indirectly, these data may reveal to what extent users feel comfortable revealing personal information to social media providers (ostensibly to enrich their interaction with the platform), without researchers having direct access to the said information.

### Limitations

One of the most important problems we faced with our study was the temporal mismatch between validated public health data and Facebook advertising data. We compared the current Facebook advertising data with public health data collected nearly a year before. This is an important shortcoming as interests can change rapidly due to many external factors that are nearly impossible to control. As we mentioned earlier, waiting to obtain the *ground truth* data may be a solution. Furthermore, we do not have data on interests within Facebook from years ago. This is, however, something available in other tools such as Google Trends or Insights.

Beside the black-box limitations discussed above, more domain knowledge is required to select more *marker* interests potentially important in tracking illnesses, and our preliminary study by no means exhausts the potential interests that could be used for this purpose. In fact, we purposefully limited the selection of interests to avoid the multiple hypotheses problem, and to focus just on the major ones. However, a fuller list of interests may be provided by the experts when studying a particular phenomenon. We found the Facebook Advertising Manager to be a useful tool in this, as it provides suggestions of interests related to ones already selected. We also must notice that taxonomies and categories of online health data, including Facebook, do not always correspond with the taxonomies of health authorities. This is a strong limitation for the integration of social media and public health data.

One more potential limitation of this study is that some users do avoid using Facebook due to privacy concerns [63]. A danger of relying on social media platforms such as Facebook for public health monitoring is that we might be excluding parts of the population that avoid such platforms due to ethical and privacy concerns. On the other hand, the high adoption of those platforms also calls for the utilization of such platforms in public health, but always considering the overall context of the health care system. Furthermore, there might be some topics of high importance in public health that are not present in Facebook due to privacy issues and socio-cultural factors (eg, family planning, sexual health, mental health). For these, studies using hybrid methodologies, which encompass resources other than social media, are necessary.

### Conclusions

In this study, we explored whether Facebook advertising audience estimates can be used to track real-world health statistics. We proposed methodological baselines, aka *placebos*, for the evaluation of these estimates, and illustrate their performance on selection of use cases. The health-related interests can be useful for the design of health-risk surveillance, health interventions recruitment, among many other applications. This study describes experimentally driven approaches to tackle the closed (aka black-box) nature of Facebook advertising, as in any social media tool, for the use in public health monitoring.

## Abbreviations

API: Application Programming Interface
CDC: Center for Disease Control and Prevention
FB: Facebook
